# Comment on: Antiviral effect of Evusheld in COVID-19 hospitalized patients infected with pre-Omicron or Omicron variants: a modelling analysis of the randomized DisCoVeRy trial

**DOI:** 10.1093/jac/dkae385

**Published:** 2024-11-05

**Authors:** Alexis Lacout, Xavier Azalbert, Corinne Reverbel, Jean-François Lesgards, Dominique Cerdan, Valère Lounnas, Gérard Guillaume, Martin Zizi, Christian Perronne

**Affiliations:** Radiodiagnostic, Surgical Medical Center of Tronquieres–Elsan, Aurillac, France; Toulouse School of Economics Alumni, Garches, France; Biochemistry, BonSens.Org, Entzheim, France; Independent Researcher and Consultant, Marseille, France; Pharmacien, Bordeaux, France; Independent Researcher, Paris, France; Rhumatologie, Paris, France; Aerendir Mobile Inc., Mountain View, CA 94040, USA; Infectious and Tropical Diseases, Paris, France

We read with great interest the Beaulieu *et al.* article.^1^ The article shows that the drug Evusheld exhibits antiviral activity, with differences between variants. We have several objections.

- We have a serious concern regarding the applicability of mechanistic mathematical modelling (MMM). In 2018, Baker *et al.*^2^ investigated the applicability of MMM in biology. Their conclusion was: ‘To provide accurate predictions, machine learning models require large amounts of data or an intensive interaction with the environment, the choice of an adequate algorithm, and the identification of inputs and outputs of interest’. Baker *et al*. also emphasize that such modelling requires skilled and trained individuals to ensure integrity. In our opinion, authors should specify how they tried to solve this challenge. Metzcar *et al*.^3^ also states: ‘The choice of analysis tool should always keep in mind the quality, size, and type of data and knowledge in light of the underlying research question’. They conclude: ‘Finally, despite the positive notion regarding mechanistic learning, certain limitations persist within both separate and combined approaches. Specifically, ethical considerations should be addressed. These may arise from data privacy, algorithmic bias, or the clinical implementation of hybrid models.’ Gyllingberg *et al*.^4^ went further in describing ‘the lost art of mathematical modelling’ and concluded that ‘of the three modelling activities—(1) formulating models; (2) analysing models; and (3) fitting or comparing models with data—inherent to mathematical biology, researchers currently focus too much on activity (2) at the cost of (1)’. The most concerning aspect of this exercise and the use of MMM is the small sample datasets from which they derive the models. It corresponds to point (1) of Gyllingberg *et al*. of model formulation. In our opinion, the study data are not powerful enough to provide a stable model. For example, in Figures S2–5, as in the others, the authors fit curves with three observed viral loads (dots) and three observed neutralizing activities (triangles). This does not respect the foundation of the law of large numbers^5^ which gives a reminder that ‘the average of a large number of independent, or nearly independent, random variables is usually close to its mean’. In the article, we have very small datasets. With 3 points, it is well below what would be adequate to have a stable and usable mechanistic model. The use of the models where a mean is calculated will therefore contain biases, which may lead to completely wrong conclusions.
- Approximately half of the patients were vaccinated. Wouldn’t vaccination interfere with viral load measurements? It is not clear whether the authors considered patients’ vaccination status when measuring viral load after treatment.
- The study was carried out on hospitalized patients. At this stage, which is the beginning of the inflammatory phase of the disease, it is no longer the antiviral effect that is of most interest from a care point of view, but nursing, oxygen therapy and the various drugs that could prevent the cytokine cascade. Viral loads are no longer clinically relevant. Antiviral drugs must be administered as early as possible, well before hospitalization, with close monitoring of oxygenation, because of the hypoxia phenomena that can occur (shunt effect). In fact, as the authors state in their article, Results section: ‘To achieve a 0.5 log difference in viral load levels at Day 5 compared with untreated patients, which was associated with a reduction in the risk of severe disease in outpatients…’. It is therefore surprising that the authors chose these hospitalized patients in the inflammatory phase to carry out this study.
- An antiviral drug is administered in the early stages of the disease, before the patient is hospitalized. The cost and IV administration of this drug mean that it cannot be used on an outpatient basis, before hospitalization. The vast majority of patients do not progress to respiratory distress. It is inconceivable to administer this drug early and systematically, even to patients with risk factors.
- The authors make no mention of the evolution of SARS-CoV-2 infection possibly aggravated by monoclonal antibodies, which could select variants that could escape the immune response. The authors could also discuss the possibility of variant-dependent antibody-dependent enhancement phenomena.^6–8^
- The authors also report a reduction in mortality with remdesivir. However, this reduction was small in the article cited, observed only in non-ventilated patients. Once again, these studies were carried out too late, and the authors make no mention of the particularly severe renal and hepatic complications of this drug.^9^

While interesting from a fundamental point of view, we see little practical application of this approach in terms of real early patient management.
