# Correction to: Comment on: Antiviral effect of Evusheld in COVID-19 hospitalized patients infected with pre-Omicron or Omicron variants: a modelling analysis of the randomized DisCoVeRy trial

**DOI:** 10.1093/jac/dkaf492

**Published:** 2026-01-28

This is a correction to: Alexis Lacout, Xavier Azalbert, Corinne Reverbel, Jean-François Lesgards, Dominique Cerdan, Valère Lounnas, Gérard Guillaume, Martin Zizi, Christian Perronne, Comment on: Antiviral effect of Evusheld in COVID-19 hospitalized patients infected with pre-Omicron or Omicron variants: a modelling analysis of the randomized DisCoVeRy trial, *Journal of Antimicrobial Chemotherapy*, Volume 80, Issue 3, March 2025, Pages 891–892, https://doi.org/10.1093/jac/dkae385.

In the originally published version, Dr Martin Zizi’s affiliations were incorrectly indicated. The correct affiliation for Dr Zizi is Aerendir Mobile Inc., Mountain View, CA 94040, USA.

This has been corrected in the published item.

